# Sulbactam combined with tigecycline improves outcomes in patients with severe multidrug-resistant Acinetobacter baumannii pneumonia

**DOI:** 10.1186/s12879-022-07778-5

**Published:** 2022-10-21

**Authors:** Yanling Deng, Lin Chen, Mingrui Yue, Xiaobo Huang, Yang Yang, Hua Yu

**Affiliations:** 1 Department of Respiratory and Critical Care Medicine, Sichuan Provincial People’s Hospital, University of Electronic Science and Technology of China, Chengdu, China; 2 Department of Intensive Care Unit, Sichuan Provincial People’s Hospital, University of Electronic Science and Technology of China, Chengdu, China; 3 Chinese Academy of Sciences Sichuan Translational Medicine Research Hospital, 610072 Chengdu, China; 4 Department of Laboratory Medcine, Sichuan Provincial People’s Hospital, University of Electronic Science and Technology of China, Chengdu, China

**Keywords:** Multi-drug resistant *Acinetobacter baumannii* (MDR-AB), Carbapenem-resistant *Acinetobacter baumannii*(CRAB), Severe pneumonia, Intensive care unit

## Abstract

**Background:**

The purpose of this study was to review the treatment plan of patients with multidrug-resistant *Acinetobacter baumannii* (MDR-AB) pneumonia and analyze the factors associated with patient deaths and the medication regimen.

**Methods:**

We collected 1,823 qualified respiratory specimens that were culture-positive for MDR-AB. 166 patients confirmed to have hospital-acquired MDR-AB pneumonia were selected as the research subjects. The differing clinical characteristics and treatment interventions between the surviving group and death group within 28 days were analyzed.

**Results:**

The mortality rate was high for those aged > 75 years (*p* = 0.001). Patients who underwent invasive catheter placement (*p* < 0.001) and mechanical ventilation (*p* = 0.046) had a higher mortality rate. Combination therapy with tigecycline can reduce the mortality rate (*p* < 0.001) of MDR-AB pneumonia in patients with carbapenem-resistant AB(CRAB). Combination therapy with sulbactam was shown to reduce the mortality rate (*p* < 0.001), and high-dose sulbactam (> 3 g/day) might be better than low-dose sulbactam (≤ 3 g/day).

**Conclusion:**

Reducing the time of invasive catheter placement and mechanical ventilation in patients in the intensive care unit (ICU), antimicrobial treatment, combined with tigecycline and sulbactam, might help reduce the mortality rate in patients with severe MDR-AB hospital-acquired pneumonia.

## Background

*Acinetobacter baumannii* (AB) is a common pathogen that causes opportunistic infections in hospitals. According to the China Antimicrobial Surveillance Network (CHINET) continuous drug resistance monitoring program, AB is the second most common gram-negative bacterium isolated from respiratory specimens and accounted for about 17.07% of infections in 2020. Important risk factors for AB infection include admission to the ICU, impaired immune function, receiving broad-spectrum antibacterial drugs, and mechanical ventilation [1,2]. Since AB has a high rate of resistance to antimicrobial drugs, the control of multi-drug resistant AB (MDR-AB) is currently one of the main goals of nosocomial infection prevention and control.

In Asia, the sensitivity of AB to carbapenems were less than 27% [3]. As such, a number of guidelines recommend the combination of polymyxin, tigecycline, carbapenems, sulbactam, or other antibacterial drugs for the treatment of MDR-AB pneumonia in China or abroad [4, 5]. However, the sensitivity of AB to certain drugs differs in different regions. Thus, it is necessary to design an appropriate combination drug regimen according to the local drug resistance situation and common guidelines and recommendations.

It is a challenge for clinicians to choose antibacterial drugs when trying to control severe MDR-AB infection, especially carbapenemase-resistant *Acinetobacter baumannii* (CRAB). According to an in vitro drug sensitivity test, the resistance rate of AB to carbapenems ranged from 62–100% [3], and the resistance rate to cefoperazone/sulbactam was 46.3% [6]. Antibiotics containing sulbactam were the main drugs for the treatment of *Acinetobacter* infection in China, partly because of the economic burden. The conventional dose is 4 g/d for MDR-AB, but it can be increased to 6.0 g/d or even 8.0 g/d according to expert consensus on the diagnosis, treatment, prevention, and control of AB infection in China [4]. In addition to sulbactam, tigecycline is the most used for the control of hospital-acquired MDR-AB, but its clinical efficacy is controversial [7]. Studies have shown that even if AB was sensitive to tigecycline in vitro, it could not improve the prognosis of patients with MDR-AB. The main objective of this study was to investigate feasible and effective treatment options for patients with severe MDR-AB pneumonia, from a clinical perspective. Here, we retrospectively identified factors influencing mortality in patients with severe MDR-AB pneumonia and appropriate antibiotic regimens, especially the combined effect of sulbactam and tigecycline.

## Methods

### Subjects

**Fig. 1:**
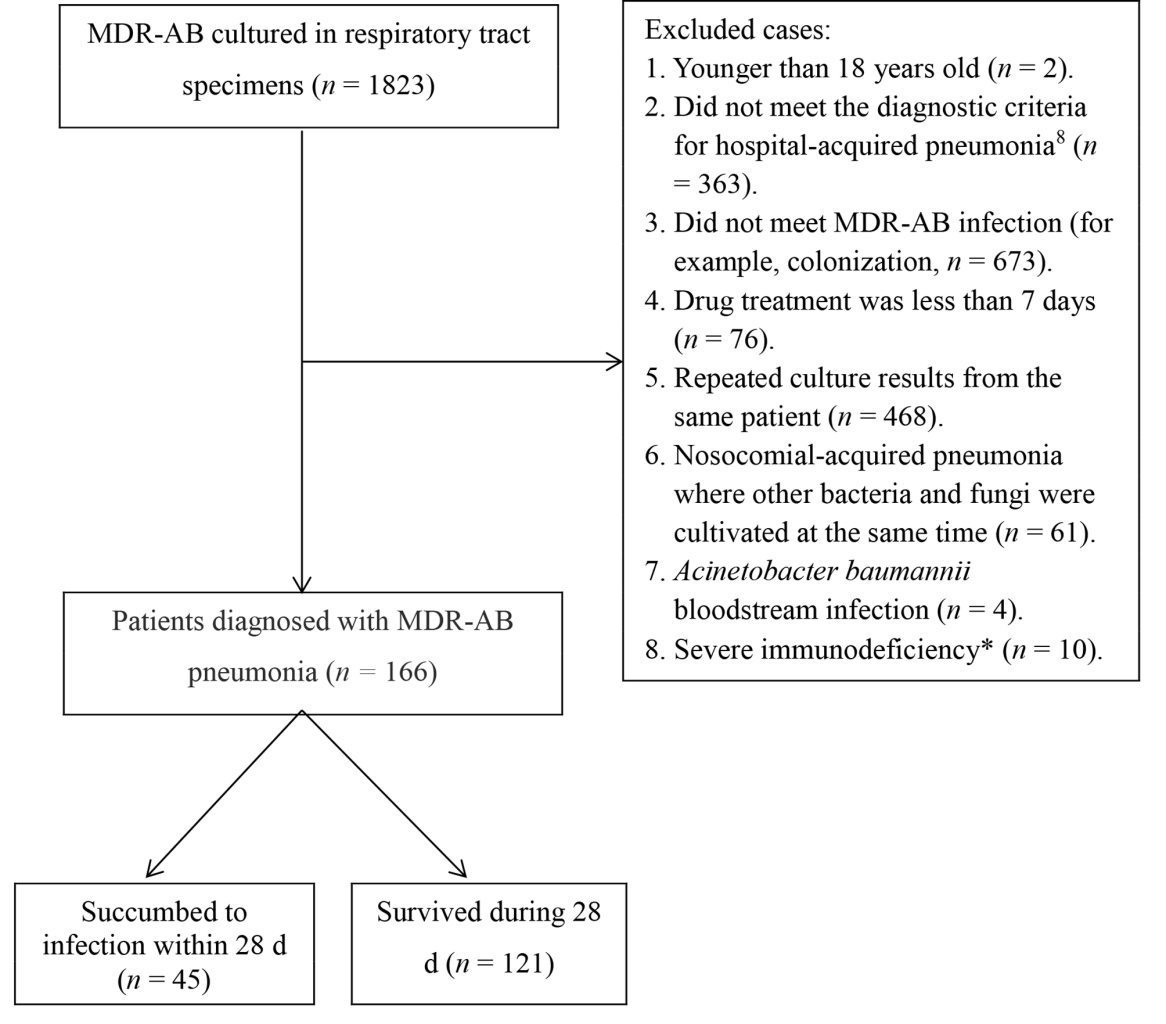
MDR-AB inclusion and exclusion flow chart. *Severe immunodeficiency patients: long-term use of immunosuppressants, solid organ transplant patients, recent use of high-dose methylprednisolone drugs (more than 280 mg/w), hematological malignancies, and patients with severe agranulocytosis. MDR-AB, Multi-drug resistant *Acinetobacter baumannii*

We enrolled 1,823 respiratory tract specimens cultured as MDR-AB from 2016 to 2020 in this study. Out of the 1,823 cases, 166 patients were diagnosed with MDR-AB pneumonia. The screening process was shown in Fig. 1. This retrospective study was approved by the ethics committee of Sichuan Provincial People’s Hospital (Affiliated Hospital of the University of Electronic Science and Technology of China). Approval number: 2019059B.

### Method for detecting carbapenemase-producing genotype

The carbapenemase detection kits (LFA) was used to detect the 157 CRAB strains, which provided by Beijing Gold Mountainriver Tech Development Co., Ltd. Take the strains out of the − 80 °C freezer 24 h in advance and wait at room temperature to thaw. Dip the mixed strain with an inoculating loop and inoculate it on a blood plate or a MacConkey plate in a four-district line. Place these plates in a constant temperature incubator at 35 °C for 18-24 h. Then, fresh strains of AB grown uniformly over 3 zones on the plates were used for the assays.

### Clinical information

For each patient, the following information was collected: age, sex, underlying diseases, initial diagnosis at admission, clinical features, laboratory findings, other microorganism expect AB, radiological findings, clinical course until diagnosis, antibiotic and invasive treatment, Acute Physiology and Chronic Health Evaluation II (APACHE II) score at admission, and in-hospital outcome.

### Treatment

Each treatment plan used to treat patients with MDR-AB was collected. Treatment plans included invasive catheterization (tracheal intubation, central venous catheterization, and urinary tract catheterization) and antibiotic use (polymyxin, tigecycline, carbapenem, sulbactam, aminoglycosides, quinolones, and tetracyclines). Preparations containing single sulbactam were divided into a high-dose group (> 3 g/day) and a low-dose group (≤ 3 g/day). Cases with delayed treatment were excluded. Delayed treatment meant that the clinician obtained the MDR-AB culture results from the laboratory but did not give effective antimicrobial treatment within 3 days.

1. Dose and duration of tigecycline: the first dose of tigecycline was 100 mg (2 bottles), and then 50 mg (1 bottle) every 12 h. The intravenous infusion time of tigecycline should be once every 12 h, about 30 ~ 60 min each time. The course of treatment in this study was 5–14 days.
2. Carbapenem uses meropenem, dose and duration: it was prepared with sterile water for injection, containing 250 mg meropenem per 5ml, the concentration was about every 50 mg/ml, and it was administered once every 8 h, 500 mg each time, intravenous drip.
3. Sulbactam preparation used single sulbactam to participate in the combined treatment, dose and duration: 2 ~ 4 g per day. The same amount was given intravenously concomitant infusion with cefoperazone once every 12 h or 8 h for 30 ~ 60 min.

### Statistical methods

SPSS 23.0 software (SPSS Inc., Chicago, United States) was used to analyze the data. Measurement-type data were tested for normality and homogeneity of variance. The data that conformed to normal distribution and homogeneity of variance were expressed as the mean ± standard deviation (*x* ± *s*) and analyzed using a *t-*test. An adjusted *t*-test method was used for the data that conformed to normal distribution but did not conform to homogeneity of variance. The data that did not conform to normal distribution were expressed as the median [M (Ql, Qu)], and the rank sum test was used for analysis. A chi-square test was used for counting data. After statistical analysis, the independent variables with statistical significance were selected, and the selected variables were subjected to multi-independent logistic regression analysis. A *p*-value < 0.05 was regarded as statistically significant.

## Results

### General clinical conditions

Of the 166 patients diagnosed with severe hospital-acquired MDR-AB pneumonia, 111 were males (Table 1). The median age was 69 years (19–94 years). We divided the patients with severe MDR-AB pneumonia into two groups based on the clinical outcome during 28 days of treatment: (1) death group (*n* = 45) and (2) survival group (*n* = 112). The death group was mainly concentrated in the age group over 75 years old (73.3%), which was consistent with our analysis. That patients older than 75 years had an increased risk of death (OR 0.921, *p* = 0.001). The median APACHE II score of the study population was 20. (The APACHE II score was used to assess the condition and prognosis of ICU patients. A score of > 15 was classified as more critically ill. The higher the score, the higher the risk of death during hospitalization.) Common underlying diseases were neurological diseases, heart diseases, and chronic lung diseases. Mechanical ventilation (OR 4.525) and invasive catheterization (OR 48.526) significantly increased the risk of death in severe MDR-AB patients (*p* < 0.05).

**Table 1:** Clinical data of patients with MDR-AB pneumonia

	Death group (*n* = 45)	Survival group (*n* = 121)	*P* value
Data			
Age (Y)	81 (72.5–85.5)	64 (50–75.5)	< 0.001
Gender	29 (64.4%)	82 (67.8%)	0.686
APACHEII Scores	20 (20, 22.5)	20 (16, 33)	0.094
Underlying diseases			
Liver diseases	7 (15.6%)	12 (9.9%)	0.31
Neurovascular disease	18 (40.0%)	53 (43.8%)	0.66
Kidney diseases	16 (35.6%)	14 (11.6%)	< 0.001
Diabetes	11 (24.4%)	16 (13.2%)	0.082
Pulmonary diseases	23 (51.1%)	37 (30.6%)	0.014
Heart disease	27 (60.0%)	41 (33.9%)	0.002
Immune abnormalities	4 (8.9%)	14 (11.6%)	0.621
Treatment			
Treatment delay	7 (15.6%)	19 (15.7%)	0.982
Mechanical ventilation	38 (84.4%)	65 (53.7%)	< 0.001
Invasive catheterization	44 (95.6%)	77 (63.6%)	< 0.001
Carbapenems	43 (95.6%)	92 (76.0%)	0.004
Tigecycline	20 (44.4%)	98 (81.0%)	< 0.001
Sulbactam	20 (44.4%)	80 (66.1%)	0.011

APACHEII, Acute Physiology and Chronic Health Evaluation II; MDR-AB, Multi-drug resistant *Acinetobacter baumannii*

### Characteristics of Acinetobacter baumannii strains(abs)

Of the 166 patients diagnosed with severe MDR-AB, 157 were confirmed to be carbapenemase-producing. OXA-23 accounted for 98.1% of the genotypes of these CRAB strains, and only 3 patients had NDM. The drug susceptibility results of all Abs in vitro were shown in Table 2.

**Table 2:** The susceptibility results of Abs in vitro

	Total (n = 166)		Death group (n = 45)		Survival group (n = 121)	
	S	I	S	I	S	I
Ampicillin/sulbactam	3/166	8/166	1/45	0/45	2/121	8/121
Piperacillin	0/166	0/166	0/45	0/45	0/121	0/121
Carbapenems*	9/166	0/166	4/45	0/45	5/121	0/121
Piperacillin/Tazobactam	1/166	3/166	0/45	0/45	1/121	3/121
Ceftriaxone	0/166	4/166	0/45	1/45	0/121	3/121
Cefotaxime	0/166	4/166	0/45	1/45	0/121	3/121
Cefepime	2/166	3/166	1/45	1/45	1/121	2/121
Cefoxitin	0/166	0/166	0/45	0/45	0/121	0/121
Gentamicin	7/166	4/166	2/45	1/45	5/121	3/121
Tobramycin	15/166	2/166	3/45	1/45	12/121	1/121
Amikacin	12/166	1/166	3/45	0/45	9/121	1/121
Ciprofloxacin	4/166	0/166	1/45	0/45	3/121	0/121
Levofloxacin	4/166	7/166	1/45	0/45	3/121	7/121
Moxifloxacin	4/166	0/166	1/45	0/45	3/121	0/121
Tetracycline	3/166	6/166	1/45	0/45	2/121	6/121
Tigecycline	161/166	5/166	44/45	1/45	117/121	4/121
Trimethoprim/Sulfamethoxazole	17/166	0/166	5/45	0/45	12/121	0/121
Cefoperazone/Sulbactam	3/166	7/166	1/45	2/45	2/121	5/121
Minocyclline	25/166	13/166	4/45	2/45	21/121	11/121
Ampicillin	1/166	0/166	0/45	0/45	1/121	0/121
Amoxicillin/Clavulanic	6/166	0/166	2/45	0/45	4/121	0/121
Aztreonam	7/166	0/166	3/45	0/45	4/121	0/121
Cefotetan	1/166	0/166	1/45	0/45	0/121	0/121
Cefazolin	0/166	0/166	0/45	0/45	0/121	0/121

*Carbapenems including Imipenem/Meropenem/Doripenem

### Antibiotic medication

All patients were prescribed 2 or more antibiotics for CRAB pneumonia. According to the recommendations of the MDR-AB diagnosis and treatment guidelines, most of the patients in the study used a combination treatment plan, and only a few were treated with one drug. Some single-agent treatments were explained in detail below (details were shown in Table 3).

**Table 3:** Antibiotic usage and clinical outcomes in patients with CRAB pneumonia. Clinical outcomes of different antibiotic regimens in CRAB pneumonia

Antibiotics	Number of cases	Number of deaths(Deaths/Total*100%)
Sulbactam + Carbapenems + Tigecycline	59	12(20.34%)
Sulbactam + Carbapenems	24	3(1.25%)
Sulbactam + Tigecycline	30	2(6%)
Carbapenems + Tigecycline	23	3(13.04%)
Sulbactam	0	0(-)
Carbapenems*	21	21(100%)
Colistin	2	1(50%)
Not using any of the above three drugs	0	0(-)

* These patients were treated with other types of antibiotics, including 12 cases with quinolones, 7 cases with other β-lactams, and 2 cases with tetracyclines

### Tigecycline

Among the 166 patients diagnosed with sever MDR-AB pneumonia, the resistance rate to tigecycline was 3% (5/166) in vitro ( Sensitivity to tigecycline was defined as MIC < 2 mg/L). There were 113 cases were prescribed tigecycline, and the 28-day mortality rate was 16.7%. The remaining 48 patients who did not use tigecycline exhibited a higher mortality rate of 53.2% (*p* < 0.001). There were another five patients were not prescribed tigecycline because of the MIC values of tigecycline higher than 2.

### Carbapenem

There were 157 cases resistant to carbapenem, of which 127 cases produced carbapenemase. These patients were diagnosed with CRAB pneumonia. The resistance rate to carbapenem was 94.6% (157/166). The enzyme-producing genotypes of these CRABs have been described previously. Except for 3 strains producing NDM, the rest were all OXA-23 type. Although the carbapenem resistance rate was high, there were still 104 cases with carbapenem included in the combination treatment regimen. Based on whether tigecycline (T) or sulbactam (S) was used, they were divided into TS group and TS unused group. As a result, the mortality rate in TS group was 6.7%; however, the mortality rate in TS unused group was 30.7% (*p* = 0.007). The main combined antibiotics in the TS unused group included quinolones (12 cases), other β-lactam antibiotics (9 cases), and tetracyclines (2 cases).

### Sulbactam

**Fig. 2:**
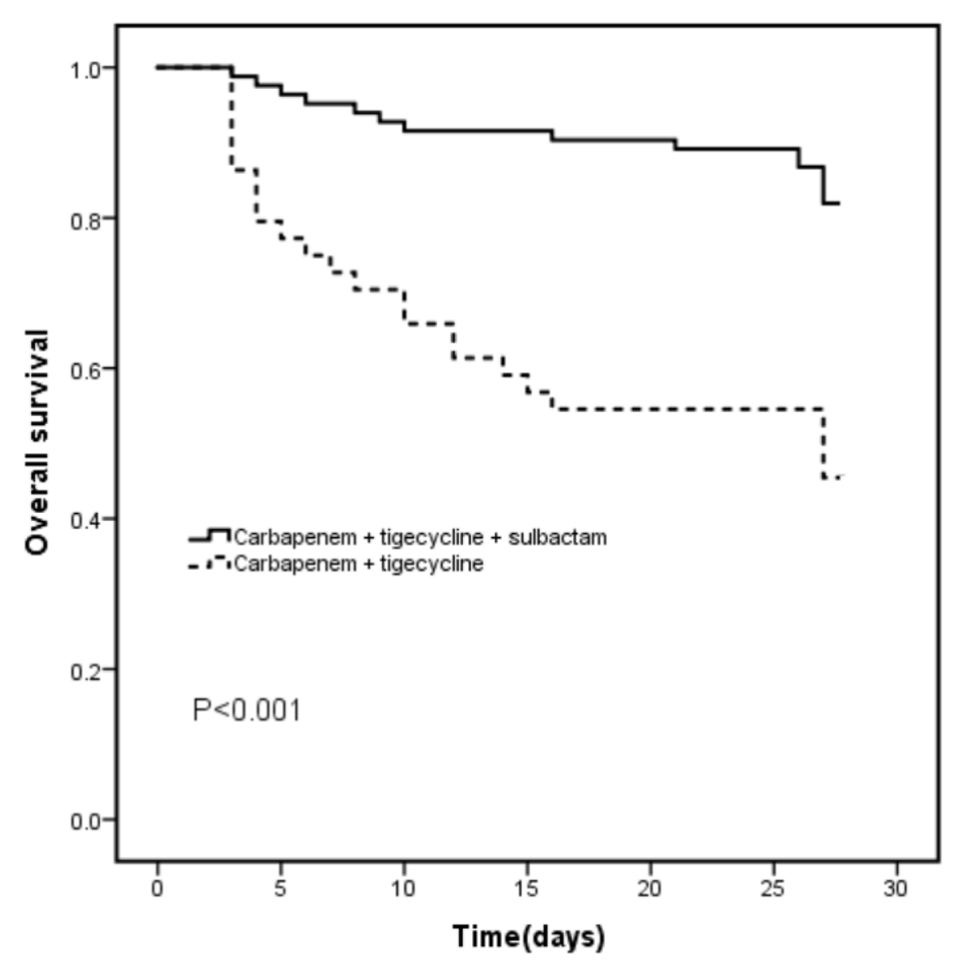
The 28-day survival rate in CRAB patients treated with Carbapenem + Tigecycline or Carbapenem + Tigecycline and Sulbactam. CRAB, carbapenem-resistant *Acinetobacter baumannii*

**Fig. 3:**
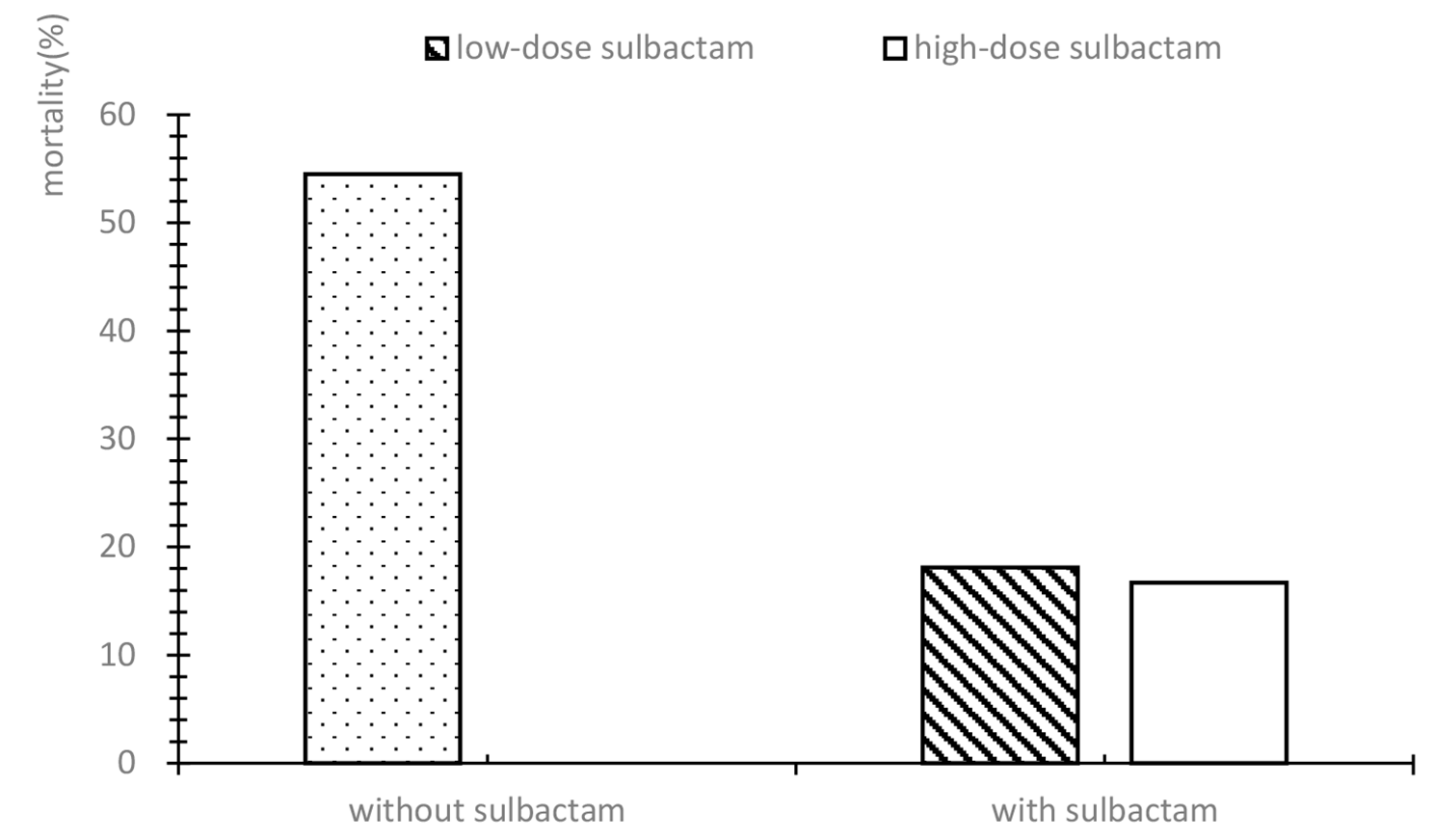
Mortality rate in patients with CRAB pneumonia treated with Carbapenem + Tigecycline alone or Carbapenem + Tigecycline and sulbactam. CRAB, carbapenem-resistant *Acinetobacter baumannii*

From the above results, sulbactam was effective in the treatment of CRAB in addition to tigecycline. The 28-day mortality of patients with CRAB pneumonia was completely different among the different combination regimens, as shown in Fig. 2. The mortality rate of the regimens that included tigecycline, carbapenem, and sulbactam was much lower than that of tigecycline plus carbapenem alone. The effect of the dose of sulbactam on the efficacy of CRAB should be further clarified. The mortality rate in CRAB pneumonia patients treated with carbapenem combined with sulbactam was 18.1%, whereas the mortality rate was 54.5% (*p* < 0.001) in those patients who were treated with the combination of carbapenem and other antimicrobial drugs (including aminoglycosides, fluoroquinolones, and tetracyclines). For CRAB pneumonia, the addition of sulbactam may reduce the MIC value of carbapenem through a synergistic effect. These patients who were given carbapenem and sulbactam in combination could be divided into a high-dose group and a low-dose group according to the sulbactam dosage. The high-dose group (30 cases) had a mortality rate of 16.7%, and the low-dose group (53 cases) had a mortality rate of 18.9% (*p* = 0.802) (Fig. 3).

### Colistin

There were only 2 patients received colistin for the treatment of CRAB. Patient 1 was automatically discharged from the hospital with colistin for less than 3 days; The second patient had delayed treatment and was diagnosed with invasive Aspergillus pulmonary pneumonia at the same time. Therefore, there were no sufficient data to be included in the evaluation of efficacy regarding colistin or combination regimens of colistin.

## Discussion

MDR-AB increases the mortality rate of nosocomial pneumonia. Our study found that admission to the intensive care unit, age > 75 years, invasive catheterization, and mechanical ventilation increased the mortality of patients with severe MDR-AB pneumonia, which was consistent with the results of other studies [9]. Early removal of invasive catheters and reducing the time on mechanical ventilation might reduce the mortality of critically ill patients diagnosed with severe MDR-AB pneumonia. Table 1 shows that there were age differences in clinical outcomes in the study population, but no differences by gender. This may be related to factors such as more complications, more co-morbidities, difficulty in weaning from ventilator, and longer ICU stay in older adults with severe pneumonia.

In our study, the main drugs that affect the mortality of patients with severe MDR-AB pneumonia or CRAB pneumonia included tigecycline, carbapenem, and sulbactam. Although Abs had good sensitivity to colistin in vitro, the MIC of colistin was less than 1 in China. But colistin was not widely used in the treatment of CRAB because of its high cost and not being covered by general health insurance in our country. There were only 2 patients were treated with colistin for CRAB, and they could not be included in the study due to uncertainty of clinical data and co-infection of fungal.

Although tigecycline was recommended as a first-line drug for CRAB pneumonia, there were different opinions on whether its use was beneficial to reduce mortality. The resistance rate of tigecycline in vitro was low in China (about 2.9%) [6], which ranges from 0.2 to 74.2% in other countries and regions [3]. The concentration of tigecycline was higher in tissue, and it has suitable antibacterial activity and was safe for use against local tissue infections. Therefore, it has been theorized that tigecycline was an effective antibiotic for the treatment of CRAB and can reduce mortality [10. A previous meta-analysis showed that patients treated with tigecycline for CRAB infection had a higher mortality rate, longer hospital stay, and lower microbial clearance rate as compared with other drugs [11]. Another prospective study demonstrated that tigecycline increases the mortality rate in patients with CRAB infection when the tigecycline MIC > 2 mg/L [12]. The results of our study showed that tigecycline could significantly reduce the mortality rate of hospital-acquired pneumonia when CRAB was the main pathogen and treatment was not delayed. Because of Abs isolated in this study was sensitive to tigecycline (Mean MIC = 1.5 mg/L) in vitro, and the therapeutic effect of tigecycline might be related to drug sensitivity. Even if these patients had higher APACHE II scores, some of them were prescribed tigecycline alone, there were still good clinical outcomes, if tigecycline was sensitive in vitro.

Most MDR-AB produce carbapenemases in previous study [13]. We also found that the resistance rate of carbapenem reached 94.6%. Continued use of carbapenem did not reduce the mortality of patients with CRAB pneumonia. It was not recommended to use only carbapenem for theses patients, but it might be effective as one of the combined treatment options, especially the MIC of carbapenem was less than 8. It was worth noting that, the combination of other drugs may reduce the carbapenem MIC value. The most common combination was sulbactam and or tigecycline in clinical practice. Carbapenem and sulbactam had been shown to have a synergistic effect and could reduce mortality [14]. Sulbactam, a β-lactamase inhibitor, has a suitable clinical therapeutic effect, and most of Abs has a low rate of resistance to sulbactam. Sulbactam enhanced the antibacterial effect of carbapenem by acting on the penicillin-binding protein. A previous study suggested that, compared with a single drug, carbapenem combined with sulbactam enhanced the antibacterial effect on CRAB in vitro and decreased the MIC value of carbapenem [15]. Our study found that although Abs was resistant to carbapenem, the 28-day mortality rate of patients after the addition of sulbactam was significantly reduced, which might be related to a mechanism by which sulbactam reduced the MIC value of carbapenem.

In addition, the combined dose of sulbactam needs to be further explored. A previous study found that when patients were given sulbactam 2 g q6h or 3 g q8h intravenously, the T > MIC (above 60%) was 98.83% and 95.59% respectively, if the sulbactam MIC was 16 µg/ml. Therefore, high-dose sulbactam was beneficial to controlling MDR-AB infection [16]. Another study recommended that a high-dose sulbactam (9 g/d) was also beneficial in controlling MDR-AB infection [17]. Our study found that the mortality rate of patients with CRAB pneumonia was lower in the high-dose sulbactam group as compared with the low-dose group, but the difference was not statistically significant. This might be related to the small number of cases in the high-dose sulbactam group. Thus, future studies regarding the dose of sulbactam need to include a larger number of cases.

Our research has many limitations. First, we followed 1,823 clinical cases with positive AB culture results. Most of the cases were excluded because clinicians were not sure whether AB was the causative bacterium of severe pneumonia or only colonization in the lower respiratory tract. Especially at the same time, other bacterial and or fungal pathogens were cultivated from the lower respiratory tract. Second, only 166 cases were identified as severe MDR-AB pneumonia. After grouping, some data results did not reach statistical differences, for example, comparison of the clinical effects of high- and low-dose sulbactam for CRAB. Then, further mechanistic studies were needed, including the paper diffusion method or the antibiotic concentration gradient method (E-test method), to detect whether there was a synergistic effect between carbapenem and sulbactam, and different resistance mechanism in these CRAB strains.

In summary, despite the above shortcomings, ICU status, an extended period of invasive catheter retention time and mechanical ventilation time, and not using the appropriate antibacterial treatment increased the mortality in patients with severe hospital-acquired MDR-AB pneumonia. Although MDR-AB was resistant to many antibiotics, most of the MDR-AB strains in western China were still sensitive to tigecycline. The inclusion of tigecycline in the combined treatment plan could significantly reduce the 28-day mortality of patients. In addition, the inclusion of sulbactam in the treatment plan could also reduce the mortality of patients with carbapenemase-producing AB pneumonia on the basis of tigecycline. High-dose sulbactam might improve the clinical prognosis of patients by reducing the MIC of carbapenem drugs especially.

## Data Availability

The data in the study are all from the clinical medical record system of Sichuan Provincial People’s Hospital in China from 2016 to 2019. All data generated or analysed during this study are included in this published article, additional specific data can be obtained from the corresponding author.

## Abbreviations

AB: Acinetobacter baumannii.
MDR-AB: multi-drug resistant AB.
CRAB: Carbapenemase-resistant Acinetobacter baumannii.
CHINET: China Antimicrobial Surveillance Network:CHINET.
ICU: Intensive care unit.
APACHE II: Acute Physiology and Chronic Health Evaluation II.
